# Cooperation between geriatricians and general practitioners for improved pharmacotherapy in home-dwelling elderly people receiving polypharmacy – the COOP Study: study protocol for a cluster randomised controlled trial

**DOI:** 10.1186/s13063-017-1900-0

**Published:** 2017-04-04

**Authors:** Rita Romskaug, Espen Molden, Jørund Straand, Hege Kersten, Eva Skovlund, Kaisu H Pitkala, Torgeir Bruun Wyller

**Affiliations:** 1 Department of Geriatric medicine, Institute of Clinical Medicine, University of Oslo, Oslo, Norway; 2 School of Pharmacy, University of Oslo, Oslo, Norway; 3 Center for Psychopharmacology, Diakonhjemmet Hospital, Oslo, Norway; 4 Department of General Practice, Institute of Health and Society, University of Oslo, Oslo, Norway; 5 Department of Research and Development, Telemark Hospital Trust, Tønsberg, Norway; 6 Norwegian National Advisory Unit on Ageing and Health, Vestfold Hospital Trust, Tønsberg, Norway; 7 Department of Public Health and General Practice, NTNU, Trondheim, Norway; 8 Mental and Physical Health, Norwegian Institute of Public Health, Oslo, Norway; 9 Department of General Practice, University of Helsinki, Helsinki, Finland; 10 Unit of Primary Health Care, Helsinki University Hospital, Helsinki, Finland; 11 Department of Geriatric Medicine, Oslo University Hospital, Oslo, Norway

**Keywords:** Polypharmacy, Inappropriate drug use, Medication review, Geriatrics, Elderly, Home-dwelling

## Abstract

**Background:**

Polypharmacy and inappropriate drug use is associated with negative health outcomes among older people. Various interventions for improving drug treatment have been evaluated, but the majority of studies are limited by the use of surrogate outcomes or suboptimal design. Thus, the potential for clinically significant improvements from different interventions is still unclear. The main objective of this study is therefore to evaluate the effect upon patient-relevant endpoints of a cooperation between geriatricians and general practitioners on complex drug regimens in home-dwelling elderly people.

**Methods:**

This is a cluster randomised, single-blind, controlled trial where general practitioners are invited to participate with patients from their lists. The patients must be 70 years or older, use at least seven different medications and have their medications administered by the home nursing service. We plan to recruit 200 patients, with randomisation at physician level. The intervention consists of three main parts: (1) clinical geriatric assessment of the patient, combined with a thorough review of their medications; (2) a meeting between the geriatrician and general practitioner, where the two physicians combine their competence and knowledge and discuss the drug list systematically; (3) clinical follow-up, depending on the medication changes that have been done. The study period is 24 weeks, and the patients are assessed at baseline, 16 and 24 weeks. The primary outcome measure is health-related quality of life according to the 15D instrument. Secondary outcome measures include physical and cognitive functioning, medication appropriateness, falls, carer burden, use of health services (hospital or nursing home admissions, use of home nursing services) and mortality.

**Discussion:**

Our choice of patient-relevant outcome measures will hopefully provide new knowledge on the potential for clinical improvements after performing comprehensive medication reviews in home-dwelling elderly people receiving polypharmacy.

**Trial registration:**

ClinicalTrials.gov, NCT02379455. Registered on 27 February 2015.

**Electronic supplementary material:**

The online version of this article (doi:10.1186/s13063-017-1900-0) contains supplementary material, which is available to authorized users.

## Background

### Polypharmacy

The drug consumption among older people has increased in the last decades [1] and polypharmacy is frequent [2]. It has been shown that 20% of Norwegians aged 70 plus are prescribed more than ten different drugs annually [3]. In a study of drug consumption among elderly (65 plus) users of home services in eight European countries, 51% used more than six and 22% used more than nine different drugs during a week [4].

A complex clinical situation involving multimorbidity may justify a high number of drugs, and a properly titrated polypharmacy can be beneficial for the individual patient. However, most therapeutic guidelines are based upon research carried out in patients with a limited number of comorbid conditions. Accordingly, such guidelines may be of limited value in frail and multimorbid elderly patients [5, 6]. The more drugs an individual patient consumes, the more demanding is the trade-off between benefits and harms, and the more tightly should the clinical condition be monitored. Polypharmacy increases the risk of adverse drug reactions (ADRs), interactions and other drug-related problems (DRPs), and the risk increases almost linearly with the number of drugs used [7–9]. Several studies among older people indicate that inappropriate drug use is a major reason for poor health and impaired function [10–12], preventable hospital admissions [13–15], and even deaths [16]. A recent Norwegian study reported that of home-dwelling elderly (75 plus) emergency-admitted to hospital, almost 40% were prescribed at least one potentially inappropriate medication (PIM) [17]. DRPs are often caused by overmedication, choice of inappropriate drugs, or inadequate monitoring and follow-up [12]. Underuse of potentially beneficial drugs is probably also a significant problem in the elderly [18, 19].

### Improving prescribing quality

Optimisation of pharmacotherapy should be based on a comprehensive assessment of all relevant medical conditions as well as the patient's functional ability, resources and preferences. However, health services are often organised in a way that makes it difficult to attain a sufficiently good overview of the clinical situation to make good medication reviews in frail and multimorbid patients. Specialists in hospitals mainly see the patient during acute exacerbations of their chronic diseases, and will naturally focus upon drugs relevant for the acute illness – especially drugs relevant for the organ system in which they have specialised. On the other side, general practitioners (GPs) are responsible for the long-term follow-up and repeat prescriptions. Because most GPs attain limited experience in handling complex health states, they may be reluctant to change medications initiated by hospital specialists [20]. Geriatricians are trained in the management of complex health conditions and polypharmacy and have firm knowledge about age-related changes in physiology and pharmacology [21], but typically see patients over a short period of time, either in an acute geriatric ward or in the geriatric outpatient department. Thus, the geriatrician often has limited information about the patient’s medical history, and has a limited possibility to follow the patient over time, elements that are typical strengths in the follow-up made by the GP. Geriatricians and GPs therefore have complementary strengths for managing complex drug regimens in frail elderly patients, and a closer cooperation between these two specialities could be beneficial.

A majority of interventions for improving drug treatment among older people are evaluated by the use of surrogate outcomes such as frequency of DRPs [22], number of prescribed drugs [23], or prevalence of PIMs [24]. Many studies also suffer from suboptimal design, such as observational studies and interventional studies without a control group [25] or without randomisation [26]. A recent Cochrane review [27] concluded that interventions to improve drug therapy appear beneficial in terms of reducing inappropriate prescribing, but the clinical benefits of such reductions are unclear. Interventional studies of measures to facilitate better prescriptions, using a scientifically sound design and at the same time utilising patient-relevant outcomes as well as outcomes capturing the effect upon the families and the health care services, are so far very rare.

### Study objectives

The aim of this study is to evaluate the effect of drug optimisation interventions resulting from a cooperation between geriatricians and GPs on clinically relevant outcome measures in home-dwelling elderly people using complex drug regimens.

## Methods/design

### Study design

**Fig. 1:**
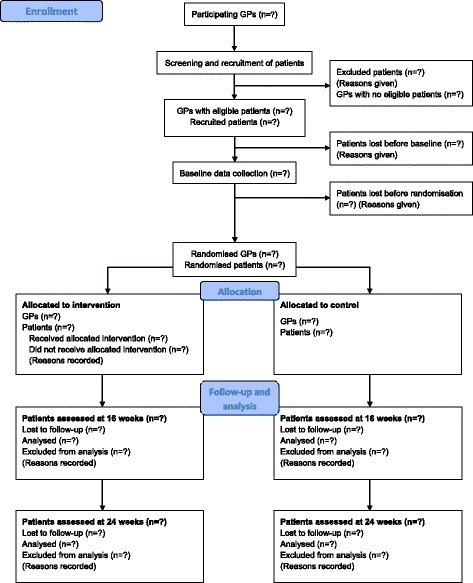
CONSORT diagram

This is a cluster randomised, single-blind, controlled trial with 24 weeks follow-up. GPs are recruited to participate in the study with patients from their lists. In order to avoid “contamination” between intervention and control patients, cluster randomisation on physician level instead of individual randomisation on patient level is performed. The outcomes are assessed at baseline and after 16 and 24 weeks. Patient flow is illustrated in Fig. 1. See Additional file 1 for the Standard Protocol Items: Recommendations for Interventional Trials (SPIRIT) checklist and Additional file 2 for the SPIRIT figure.

### Recruitment of GPs

GPs from the counties of Akershus and Oslo, Norway, are invited to participate in the study. All GPs in these areas are eligible for participation. The GPs receive written invitations, followed by a phone call to clarify if they are interested. When possible, information about the study is also given at GP meetings within each municipality.

### Study population

We assume that the comprehensive clinical evaluation and medication review that we will test is most relevant for the oldest and most frail patients, with relatively pronounced polypharmacy. We have therefore chosen the following inclusion and exclusion criteria:

#### Patient inclusion criteria

- Listed with one of the participating GPs
- Home-dwelling
- Medications administered by the home nursing service
- Age 70 years or more
- Use of at least seven different systemic medications taken regularly (preparations for inhalation, vitamin supplements and laxatives are included, but not topical drugs like eye drops and ointments)
- Signed informed consent given by the patient or his/her closest proxy

#### Patient exclusion criteria

- Expected to become permanently institutionalised within 6 months
- Life expectancy judged to be 6 months or less
- Moderate/severe dementia (i.e., Clinical Dementia Rating Scale (CDR) score > 1) and contact with the closest proxy less than once every other week
- Not speaking or understanding Norwegian
- The GP does not want the particular patient to participate (in case of important reasons not covered by the other exclusion criteria)

### Screening and inclusion

The majority of home-dwelling patients with medications administered by the home nursing service have their medications prepared by multi-dose packaging systems delivered by a pharmacy. Medication lists from the pharmacy are screened by the home nursing service or by GP office staff to identify patients fulfilling the inclusion criteria. The GPs then consider the eligibility of their patients based on the defined exclusion criteria.

Patients eligible for participation are contacted by the home nursing service or the GP’s office, explaining the study and asking whether the researchers may contact them. If this is accepted, the patients receive a home visit from a research assistant who gives complementary oral and written information. Informed written consent is obtained from all participants or from their closest relative in cases where the patient is unable to give a valid consent due to reduced cognitive functioning.

### Randomisation and blinding

In order to avoid large variation in cluster sizes, each GP can participate with a maximum of five patients. To assure as equal group sizes as possible, the GPs are stratified based on the number of contributing patients; 1–2 patients versus 3–5. Randomisation is computer-generated and carried out in blocks of unknown and variable size. The allocation sequence is prepared by a statistician not involved in recruitment, and is made available to the researchers in sealed, opaque envelopes. In order to minimise the risk of selection bias, randomisation takes place after inclusion of all patients within each cluster. The GPs are included sequentially according to the capacity in the project. The research assistant, who provides all the assessments, is blinded with respect to allocation. Given the nature of the intervention, it is not feasible to blind the patients, their relatives or the GPs. The effects of the intervention will be analysed blindly, i.e. with the two randomised groups denominated “A” and “B” but without knowledge regarding which group is the control and which the intervention group.

### Intervention

Our intervention consists of three main parts: clinical geriatric assessment of the patient combined with a thorough review of their medications; a targeted meeting between the geriatrician and the GP; and clinical follow-up.

#### Geriatric assessment and medication review

The patients receive a home visit by a physician trained in geriatric medicine (RR). In advance, the geriatrician obtains necessary information on the patient’s medical history and actual medication from hospital records, the GP’s electronic patient record, the home nursing service and other relevant sources. The geriatrician carries out a medical history from the patient (if necessary supplemented by a close relative) and a physical examination, both with focus on conditions most relevant for the patient’s total medication use. Relevant blood analyses and other supplementary tests are ordered if not already available (ECG, haematological tests, electrolytes, renal function, natriuretic peptides, thyroid function, nutritional indicators, serum concentration of relevant drugs, pharmacogenetic testing etc.). The geriatric work-up is aimed at evaluating whether current medications are indicated, whether the relevant conditions are satisfactorily compensated, whether the dosages are appropriate, whether the patient has symptoms of ADRs, and whether drug-drug interactions or drug-disease interactions are present or likely to occur. A drug interaction database [28], lists of anticholinergic drugs [29, 30], the STOPP/START criteria [31] and the NORGEP criteria [32] are also used. The geriatrician is provided with clinical supervision from a senior consultant in geriatric medicine (TBW).

#### Meeting between the geriatrician and GP

The main purpose of this meeting is to combine the competence and knowledge of the geriatrician with that of the GP. The geriatrician summarises the findings from the geriatric assessment and medication review, and the two physicians discuss the patient’s drug list systematically. The geriatrician may suggest changes in the drug regimen, but the GP retains the medical responsibility for the patient, and is in charge of all ordinations and medication changes.

#### Clinical follow-up

Depending on medication changes that have been done, the two physicians arrange the necessary follow-up within the project period. The follow-up can consist of a clinical evaluation, further drug adjustments, blood tests etc., and can be carried out by the GP, the geriatrician or through telephone contact with the patient, the relative or the home nursing service, depending on the circumstances.

### Control group

The control group receives “usual care” from their GPs during the study period, but the GPs in the control group are offered our assistance in performing medication reviews after the study period is completed.

### Outcome measures

Follow-up occurs at 16 and 24 weeks (±2 weeks) after baseline registrations, in order to study the course of any change over time.

#### Primary outcome measure

The primary endpoint is change in health-related quality of life (HRQoL), measured by the 15D instrument (single index version) at 16 weeks. 15D is a generic, 15-dimensional instrument concerning different aspects of HRQoL [33, 34] that has been used in similar geriatric interventions [35, 36]. The dimensions are mobility, vision, hearing, breathing, sleeping, eating, speech, elimination, usual activities, mental function, discomfort and symptoms, depression, distress, vitality and sexual activity. Each dimension is rated on an ordinal scale with five levels, and the respondent chooses the level best describing his/her present health status. The 15D can be used both as a profile measure and as a single index. The single index, representing the overall HRQoL, varies between 0 (poorest HRQoL) and 1 (excellent HRQoL), and is calculated by using a set of population-based preference or utility weights [33, 37]. Usually, the 15D questionnaire is filled in by the individual whom it concerns, but it is also validated for proxy raters.

We hypothesise that most improvements in the total drug regimen of frail older patients, such as better pain control, better symptom control in heart failure, less parkinsonian side effects, less iatrogenic dehydration or less sedation, have the potential to improve HRQoL. Accordingly, in our opinion 15D is an appropriate outcome measure when the aim is to improve the total drug regimen in an individualised manner across a broad spectrum of drug classes within a heterogeneous group of older users of multiple drugs. The patients included in our study are old, and many are not familiar with self-administration of questionnaires of this kind. 15D is therefore administered by interview. This is done by the research assistant, blind to group allocation. If the patient has a moderate or severe dementia (CDR > 1), and/or the research assistant considers that they do not understand the questionnaire, the interview is carried out with the closest proxy. To account for patients who might lose their ability to respond to 15D during the follow-up period, and in order to compare the answers given by patients themselves and their proxies, we administer 15D to the closest proxy in all cases.

#### Secondary outcome measures

Secondary endpoints are on the patient, family and local community level.

1. Patient-related endpoints
  15D after 24 weeks
  Short Physical Performance Battery (SPPB), a simple test of mobility that combines the results of walking speed, chair stand and balance tests [38]
  Gait speed [39]
  Hand grip strength (hand dynamometry)
  Functional Independence Measure (FIM), a measure of physical and cognitive disability [40]
  Trail Making Test A and B, measuring processing speed, focused and split attention, and executive functioning [41]
  “Digit Span”, a digit repetition test of working memory, measuring attention [42]
  Five Digits Test, measuring attention and executive functions [43]
  Appropriateness of current prescribing as assessed by the Medication Appropriateness Index (MAI) [44] and the Assessment of Underutilization (AOU) [45]
  Number of falls during the follow-up period
  Orthostatic blood pressure
  Weight
  All changes in the pharmacotherapy taking place during the intervention and follow-up period
  Mortality
2. Family-related endpoints
  Carer burden according to the Relative Stress Scale (RSS) [46]
3. Endpoints related to the local community and use of health services
  Hospital admissions (with reasons)
  Number of days the patient has spent in his or her own home (in contrast to being in hospital, nursing home or other institutions)
  Admission to permanent institutional care
  Current use of home nursing service (hours per week)

### Background variables

The outcomes are measured at baseline, in order to check and adjust for possible inequalities. In addition, the following descriptive variables are registered:

- Demographic data
- Diagnoses according to ICD-10
- Cumulative Illness Rating Scale (CIRS) [47]
- Clinical Dementia Rating Scale (CDR) [48]
- Course of cognitive symptoms during the last 10 years, according to the Informant Questionnaire on Cognitive Decline in the Elderly (IQCODE) [49]
- Nutritional status, assessed by the Mini Nutritional Assessment Short Form (MNA-SF) [50]
- Current drug use

### Data collection

Background information on diagnoses and comorbidity are obtained from the GP’s electronic patient records. The patient receives three home visits from the research assistant; at baseline, 16 and 24 weeks (±2 weeks). These visits take place where the patient is living at that moment; this might be the patient’s own home, a nursing facility or a rehabilitation institution. All assessments directly involving the patient are performed at these visits. Proxy information is collected through telephone calls and/or questionnaires sent by mail, if the proxy is not present at the home visit. Updated drug lists are obtained at all assessment points. The MAI [44] and AOU [45] are assessed by clinical pharmacists and geriatricians not involved in the intervention. The use of home nursing service and admissions to hospital, nursing home or other institutions is registered. In case of hospital admissions, the discharge summary is obtained. See Table 1 for study assessment procedures and timetable.

**Table 1:** Study assessment procedures and timetable

	Baseline visit	16 weeks follow-up	24 weeks follow-up
Assessments directly involving the patient			
Demographics, diagnoses, CIRS, MNA-SF	x		
15D	x	x	x
SPPB + gait speed	x	x	x
Hand grip strength	x	x	x
Trail Making Test A + B	x	x	x
Five Digits Test	x	x	x
Digit Span	x	x	x
Falls		x	x
Orthostatic blood pressure	x	x	x
Weight	x	x	x
Assessments of drug use			
Current drug use and changes in pharmacotherapy	x	x	x
MAI	x	x	x
AOU	x	x	x
Assessments based on observation and/or proxy information			
FIM	x	x	x
CDR	x		
Assessments based on information from a close relative			
15D	x	x	x
RSS	x	x	x
IQCODE	x		
Administrative data			
Hospital admissions (with reasons)		x	x
Number of days in own home		x	x
Admission to permanent institutional care		x	x
Use of home nursing service (hours per week)	x	x	x
Mortality		x	x

*Abbreviations*: *CIRS* Cumulative Illness Rating Scale, *MNA-SF* Mini Nutritional Assessment Short Form, *SPPB* Short Physical Performance Battery, *MAI* Medication Appropriateness Index, *AOU* Assessment of Underutilization, *FIM* Functional Independence Measure, *CDR* Clinical Dementia Rating Scale, *RSS* Relative Stress Scale, *IQCODE* Informant Questionnaire on Cognitive Decline in the Elderly

### Sample size, statistical power and statistical analysis

The primary endpoint 15D is on an interval scale, and is expected to be reasonably normally distributed. We will analyse this measure by ANCOVA, as recommended by Vickers and Altman [51]. Other outcome measures will be analysed by ANCOVA (continuous data) or logistic regression (categorical data) as appropriate. Non-normally distributed variables will be transformed in order to try to achieve a distribution that is more feasible for analysis. Robust estimation of standard errors will be used to handle within-cluster (physician) correlation. A detailed statistical analysis plan will be developed that will detail imputation processes for missing data etc.

The number of patients in the intervention group is planned to be approximately 100, and each GP can participate with 1–5 patients. This means that the number of GPs (clusters) will be 20–100 in the intervention group. A similar number of patients and GPs will be included in the control group.

It is difficult to make valid assumptions on the correlation between patients within each cluster. In order to estimate the power of the study, we have chosen to estimate power in a worst case (perfect correlation) and a best case (no correlation) scenario. The true correlation is expected to be much closer to the latter, as the potential for intervention will vary between the individual patients. Based on previous studies using 15D, the standard deviation of change over time is expected to be between 0.07 and 0.08 [35, 36, 52]. The minimum important change (MIC) for the change in 15D scores is ± 0.015 [53]. A change of more than 0.035 in the negative direction represents “much worse HRQoL” and a change of more than 0.035 in the positive direction “much better HRQoL” [53]. Based on previous studies, in addition to a pilot study, we believe that our intervention is extensive enough to potentially improve the patients HRQoL to “much better” (>0.035). As can be seen from Table 2, the power to detect a difference of 0.035 will be in the range 59 to 94%, and most probably > 80%.

**Table 2:** Estimation of power in different scenarios

Δ	SD	r	Power %
0.035	0.08	1	59
0.035	0.08	0	87
0.035	0.07	1	71
0.035	0.07	0	94
0.025	0.07	1	43
0.025	0.07	0	71

Δ change in 15D HRQoL index score, *SD* standard deviation of change over time in 15D score, *R* correlation between patients within each cluster

## Discussion

Polypharmacy and inappropriate drug use constitute a major health risk for elderly patients. On the other side, comorbid conditions and bothersome symptoms may give good reasons to prescribe many drugs. The challenge is to personalise the polypharmacy and to find the best combination of drugs for each individual patient. For this purpose, thorough clinical evaluations and close monitoring of the patient’s condition is necessary.

When designing the COOP study, we emphasised examination of real-life scenarios. Our intention is to individualise the treatment, but at the same time to evaluate the intervention with stringent scientific methods. In our opinion, the project is innovative in improving cooperation between hospital specialists and the primary health care system and evaluating the effect with patient-related outcome measures.

A majority of previous interventions for improving drug treatment among older adults have been evaluated by the use of surrogate outcomes, such as the number of prescribed drugs [23] or prevalence of PIMs [54], but this does not necessarily indicate whether the patient has really benefited from the intervention. We have chosen patient-relevant outcome measures, and will hopefully be able to contribute with valuable knowledge about clinical effects of comprehensive medication reviews.

There are some limitations to the study. The participants will be old and multimorbid, and prone to experience new illnesses and clinical deterioration during the study period, regardless of the intervention. This might make the intervention less powerful. It is also possible that the GPs who choose to participate are particularly interested in drug treatment in the elderly, and therefore have more knowledge and awareness about this topic than GPs that do not participate. The participating patients might therefore be better followed up beforehand, making it less likely that the intervention will give any effects.

The patients and their relatives are not blinded. There is a risk that the research assistant might be unblinded, even if it is clearly explained to the participants that they should not reveal their group allocation. Nor are the GPs blinded, and the GPs in the control group may pay extra attention to the drug treatment for their patients during the study period, even if they are not provided with advice from the geriatrician.

Since our aim is to address the various comorbidities and clinical problems that are unique for each participating patient, the intervention is not completely standardised. Successful interventions for geriatric patients have often been of a complex type [55–57], whereas the implementation of one single measure will be less likely to give an effect. A major challenge when studying complex interventions is to describe the intervention with sufficient precision as to facilitate replication [57]. Our main strategy for this will be to compensate for the necessary degree of pragmatism in the interventional approach with a detailed description of the interventions that were in fact carried out, in particular changes in the drug regimens of the individual patients.

This is quite an extensive intervention, but if it is effective, we will argue that it can be carried out for those patients having the most complex polypharmacy – within the existing framework of a geriatric outpatient clinic.

### Trial status

The first patient was included on 17 March 2015. The study is ongoing.

## Additional files

Additional file 1: SPRIT 2013 checklist: recommended items to address in a clinical trial protocol. (PDF 58 kb)

Additional file 2: SPIRIT figure. (PDF 15 kb)

## Abbreviations

ADRs: Adverse drug reactions
AOU: Assessment of Underutilization
CDR: Clinical Dementia Rating Scale
CIRS: Cumulative Illness Rating Scale
DRPs: Drug-related problems
FIM: Functional Independence Measure
GP: General practitioner
HRQoL: Health-related quality of life
IQCODE: Informant Questionnaire on Cognitive Decline in the Elderly
MAI: Medication Appropriateness Index
MIC: Minimal important change
MNA-SF: Mini Nutritional Assessment Short Form
PIM: Potentially inappropriate medications
RSS: Relative Stress Scale
SPIRIT: Standard Protocol Items: Recommendations for Interventional Trials
SPPB: Short Physical Performance Battery
