# Supplementary material for: Cooperation between geriatricians and general practitioners for improved pharmacotherapy in home-dwelling elderly people receiving polypharmacy – the COOP Study: study protocol for a cluster randomised controlled trial
Source: Trials. 2017 Apr 4;18:158. doi: 10.1186/s13063-017-1900-0 (PMC5379709; doi:10.1186/s13063-017-1900-0)
Supplement: Supplementary file 2 — SPIRIT figure. (PDF 15 kb) [file 13063_2017_1900_MOESM2_ESM.pdf]

	STUDY PERIOD				
	Enrolment	Allocation	Post-allocation		Close-out
TIMEPOINT	$-t_1$	0	Intervention	16 weeks	24 weeks
**ENROLMENT:**					
**Eligibility screen**	X				
**Informed consent**	X				
**Allocation**		X			
**INTERVENTIONS:**					
**Intervention group**			X		
**Control group**					
**ASSESSMENTS:**					
Background variables		X			
Primary outome measure		X		X	X
Secondary outcome measures		X		X	X
